# Effects of evidence-based strategies to reduce the socioeconomic gradient of uptake in the English NHS Bowel Cancer Screening Programme (ASCEND): four cluster-randomised controlled trials

**DOI:** 10.1016/S0140-6736(15)01154-X

**Published:** 2016-02-20

**Authors:** Jane Wardle, Christian von Wagner, Ines Kralj-Hans, Stephen P Halloran, Samuel G Smith, Lesley M McGregor, Gemma Vart, Rosemary Howe, Julia Snowball, Graham Handley, Richard F Logan, Sandra Rainbow, Steve Smith, Mary C Thomas, Nicholas Counsell, Steve Morris, Stephen W Duffy, Allan Hackshaw, Sue Moss, Wendy Atkin, Rosalind Raine

**Affiliations:** a Department of Epidemiology and Public Health, University College London, London, UK; b Department of Applied Health Research, University College London, London, UK; c Cancer Research Centre UK and University College London Cancer Trials Centre, University College London, London, UK; d Department of Biostatistics, King's Clinical Trials Unit, Institute of Psychiatry, Psychology and Neuroscience, King's College London, London, UK; e NHS Bowel Cancer Screening Programme Southern Hub, Royal Surrey County Hospital NHS Foundation Trust, Guildford, Surrey, UK; f Royal Surrey County Hospital NHS Foundation Trust, Guildford, and University of Surrey, Guildford, UK; g Wolfson Institute of Preventive Medicine, Queen Mary University of London, London, UK; h Department of Surgery and Cancer, Imperial College London, London, UK; i NHS Bowel Cancer Screening Programme North East Hub, Gateshead Health NHS Foundation Trust, Queen Elizabeth Hospital, Gateshead, UK; j NHS Bowel Cancer Screening Programme Eastern Hub, Nottingham University Hospitals, Nottingham, UK; k NHS Bowel Cancer Screening Hub, London Hub, Northwick Park and St Mark's Hospital, Harrow, Middlesex, UK; l NHS Bowel Cancer Screening Programme Midlands and North West Hub, University Hospitals Coventry and Warwickshire NHS Trust, Hospital of St Cross, Rugby, UK

## Abstract

**Background:**

Uptake in the national colorectal cancer screening programme in England varies by socioeconomic status. We assessed four interventions aimed at reducing this gradient, with the intention of improving the health benefits of screening.

**Methods:**

All people eligible for screening (men and women aged 60–74 years) across England were included in four cluster-randomised trials. Randomisation was based on day of invitation. Each trial compared the standard information with the standard information plus the following supplementary interventions: trial 1 (November, 2012), a supplementary leaflet summarising the gist of the key information; trial 2 (March, 2012), a supplementary narrative leaflet describing people's stories; trial 3 (June, 2013), general practice endorsement of the programme on the invitation letter; and trial 4 (July–August, 2013) an enhanced reminder letter with a banner that reiterated the screening offer. Socioeconomic status was defined by the Index of Multiple Deprivation score for each home address. The primary outcome was the socioeconomic status gradient in uptake across deprivation quintiles. This study is registered, number ISRCTN74121020.

**Findings:**

As all four trials were embedded in the screening programme, loss to follow-up was minimal (less than 0·5%). Trials 1 (n=163 525) and 2 (n=150 417) showed no effects on the socioeconomic gradient of uptake or overall uptake. Trial 3 (n=265 434) showed no effect on the socioeconomic gradient but was associated with increased overall uptake (adjusted odds ratio [OR] 1·07, 95% CI 1·04–1·10, p<0·0001). In trial 4 (n=168 480) a significant interaction was seen with socioeconomic status gradient (p=0·005), with a stronger effect in the most deprived quintile (adjusted OR 1·11, 95% CI 1·04–1·20, p=0·003) than in the least deprived (1·00, 0·94–1·06, p=0·98). Overall uptake was also increased (1·07, 1·03–1·11, p=0·001).

**Interpretation:**

Of four evidence-based interventions, the enhanced reminder letter reduced the socioeconomic gradient in screening uptake, but further reducing inequalities in screening uptake through written materials alone will be challenging.

**Funding:**

National Institute for Health Research.

## Introduction

Colorectal cancer is the fourth most common cause of cancer death worldwide,^1^ and the second most common in the UK.^2^ Screening by testing for occult blood in stools reduces mortality.^3^ In England, an organised colorectal cancer screening programme, the Bowel Cancer Screening Programme (BCSP), began in 2006, and offers guaiac faecal occult blood testing (gFOBT) every 2 years for people aged 60–74 years (previously up to 69 years).

The UK cancer screening programmes are run by the National Health Service (NHS) with no financial costs to participants. This approach minimises inequity in the delivery of screening, but uptake for all the programmes shows a gradient by socioeconomic status.^4^, ^5^ The strongest gradient is for colorectal cancer screening: from the first 2·6 million gFOBT invitations in 2006–09, uptake was 61% in the least deprived quintile of residential areas and only 35% in the most deprived quintile.^6^

Proposed explanations for reduced uptake of screening in more deprived groups include factors such as stress, low social support, and competing life demands.^7^ These factors are difficult to address through the screening programme. Literacy might also play an important part in uptake because information is delivered entirely through mailed written communications.^8^ Eligible adults are sent an invitation letter from their nearest regional screening hub, accompanied by a 13-page information booklet that covers complex issues such as risks and benefits (numerical information) that are designed to facilitate informed decision making. Comprehension of the screening offer, therefore, might be challenging in the most deprived areas in England, where up to half of people are either functionally illiterate or have only basic literacy and struggle with statistics.^9^

**Research in Context**

**Evidence before this study**

We searched the Ovid MEDLINE, PsycINFO, and Embase databases for reports on randomised controlled trials assessing interventions to increase uptake of cancer screening, published between 1980 and 2014, and addressing socioeconomic status. We used the search string “Neoplasm/ OR cancer OR neoplas* OR onco* OR carcinoma AND Mass screening/ OR screen* OR test OR detect* OR mass screening OR cancer screening AND Intervention studies/ OR intervention stud* OR stud* OR strateg* OR promot* OR initiative* OR behavio* OR behavio* change AND Patient acceptance of health care/ OR patient compliance/ OR attend* OR uptake OR utili?* OR particip* OR complian* OR accept* OR adher* AND breast neoplasms/ OR breast OR mammogra* OR uterine cervical neoplasms/ OR cervical OR cervix OR colorectal neoplasms/ OR colorectal OR bowel OR colon OR rectal OR CRC AND Healthcare Disparities/ OR Health Status Disparities/ OR disparit* OR education OR social class OR social status OR depriv* OR income OR socioeconomic OR socio economic”. The search retrieved 103 articles addressing socioeconomic inequality, but none used reduction of inequality as the primary endpoint.

**Added value of this study**

We identified no previous studies that used a randomised controlled design to test the efficacy of interventions designed specifically to reduce the socioeconomic status gradient in screening uptake without compromising overall uptake. Our trials assessed different ways to increase the visibility and salience of a national colorectal cancer screening programme, for which invitations are mailed every 2 years to all people aged 60–74 years in England. Because the goal was to identify interventions that could be easily implemented, all the trials were embedded in the routine call and recall system of the English Bowel Cancer Screening Programme (BCSP). This approach also eliminated any selection bias associated with a research study, and provided access to objective data on screening uptake. All four interventions were supported by evidence of efficacy in other contexts or pilot data obtained by our group. The gist and narrative leaflets, designed to provide information understandable for people with poor literacy, had no effects. By contrast, invitation letters showing general practice endorsement and enhanced reminder letters improved overall uptake. Enhanced reminder letters also significantly reduced the socioeconomic status gradient. The negative results with the leaflets might have been due to the increase in the total mass of information in the mailing. The changes that could be made to the official letters were limited. The effects of the enhanced reminder letter might, therefore, have been due to increased visibility because the reminder mailing included only one letter. These results highlight the importance of doing trials in a real-life context to discover what can be achieved with minimum intervention.

**Implications of all the available evidence**

Inequality in uptake is an important limitation of cancer screening programmes, even with systems that ensure equitable delivery of invitations or with home-based screening tests that avoid the barrier of clinic attendance. The main finding from the four ASCEND trials is that reduction in inequality is extremely difficult with use of downstream approaches in an organised screening programme. The significant reduction in socioeconomic status gradient achieved with the enhanced reminder letter might lead to improved equity at no further cost. Additionally, we found a high level of general practice support for the endorsement trial, which raised overall uptake, and, therefore, adding such endorsement to the reminder letter might increase this effect further. The negative results with two of our interventions highlight the challenges of communicating effectively with people with poor literacy who need to make decisions based on medical information.

The screening hubs are required to use the standard information booklet, but each can provide a limited amount of extra material to improve presentation of the screening offer. Additionally, a few words can be added to the standard invitation and reminder letters. The ASCEND project was designed to test four different supplements to the screening information materials aimed at modifying inequality in screening uptake. Various studies have aimed to increase cancer screening uptake, but have been done across the whole population^10^, ^11^, ^12^ or in low-income groups,^13^ whereas ASCEND was specifically designed to assess the socioeconomic status gradient. The interventions tested in ASCEND had a strong theoretical rationale for use based on evidence of improving screening uptake in low socioeconomic status groups.^14^, ^15^ The materials developed for two of the trials had also been pretested in the early stages of the programme for effects on understanding and motivation.^16^, ^17^, ^18^, ^19^, ^20^ The four trials were embedded within the routine delivery of the screening programme. The protocol of this trial is available on the trial website.

## Methods

### Study design and population

**Figure 1:**
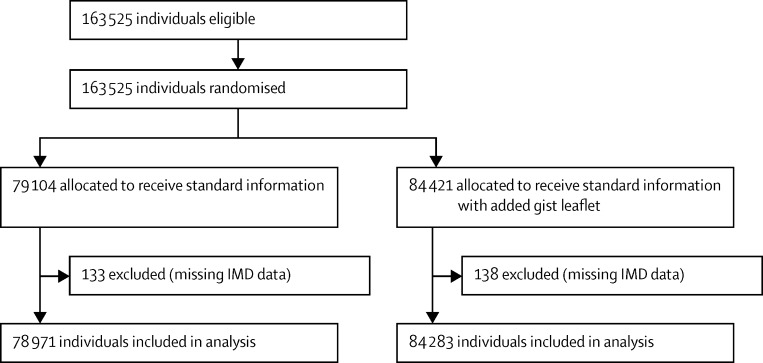
Trial 1 profile (gist leaflet) IMD=Index of Multiple Deprivation.

**Figure 2:**
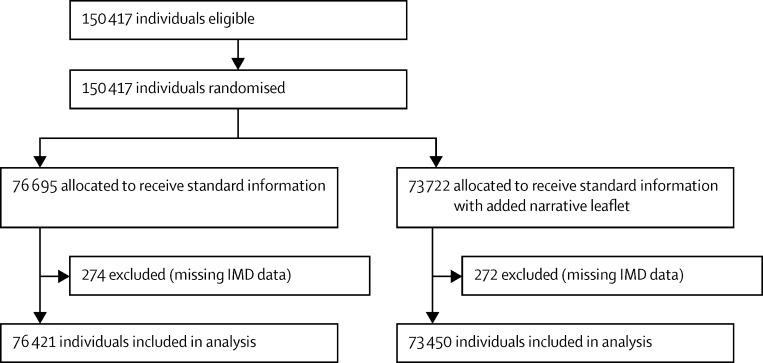
Trial 2 profile (narrative leaflet) IMD=Index of Multiple Deprivation.

**Figure 3:**
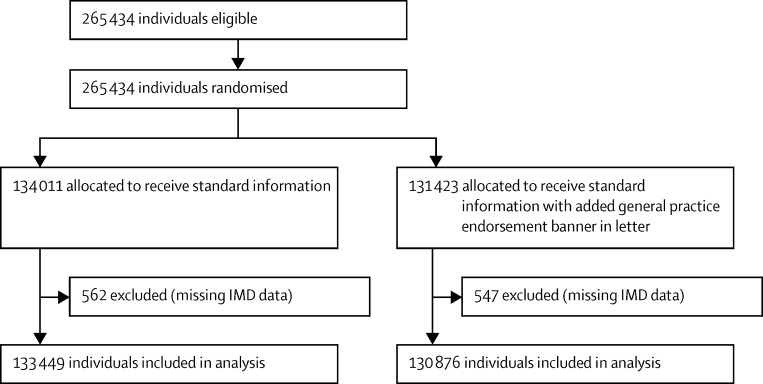
Trial 3 profile (general practice endorsement) IMD=Index of Multiple Deprivation.

**Figure 4:**
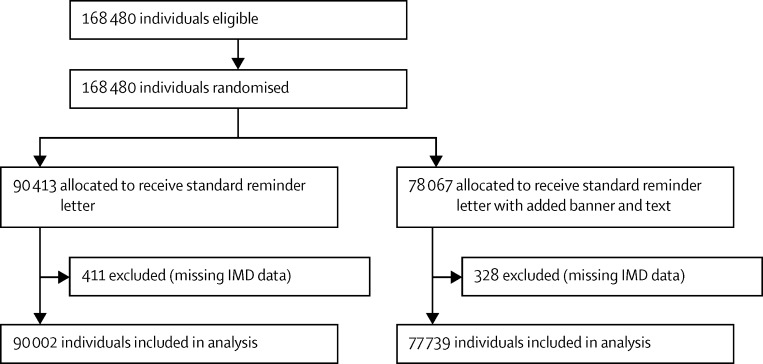
Trial 4 profile (enhanced reminder letter) IMD=Index of Multiple Deprivation.

We did four separate, two-arm, cluster-randomised controlled trials that involved individuals eligible to receive routine invitations from the BCSP for screening (Figure 1, Figure 2, Figure 3, Figure 4). The trial designs used time-defined cluster randomisation to usual care (standard information and letter [control]) or the intervention (supplemented information or letter). The interventions were designed to be implemented within the routine procedures of the BCSP, which are covered by Health and Social Care Information Centre (HSCIC) approval in relation to patient-identifiable data. We obtained ethics approval for all trials from the National Research Ethics Service Committee London-Harrow.

At any time, about 9 million adults in England are eligible for gFOBT through the BCSP. The programme is coordinated by five regional screening hubs. All five hubs were included in the study. People in each region and registered with a general practitioner are eligible for screening from age 60 years, and biennially thereafter up to and including age 74 years. Unless individuals have explicitly opted out of screening, all eligible people are sent invitation letters and screening information by their regional hub. Thus every individual scheduled to be invited during the study periods was eligible for inclusion.

8–10 days after the initial invitation letter, recipients are sent gFOBT kits and instructions. Individuals are asked to collect two samples from each of three separate bowel motions, and to return the completed kit to the regional hub, in a prepaid envelope, for processing. If kits are deemed to be spoilt, have a technical failure, or yield an unclear result, a repeat gFOBT kit is sent. If a recipient does not respond, a reminder letter is sent 4 weeks from the time of the initial invitation. If there is no response after a further 13 weeks, the individual's “screening episode” is closed for that period. If gFOBT yields an abnormal result, the person is referred to his or her local screening centre for diagnostic investigations.

### Trial 1: gist leaflet

The gist leaflet (appendix) was a simplified version of the screening information designed to be understood by readers with low literacy, numeracy, or both. This approach was informed by psychological theory^21^ and evidence gained predominately in the USA and in groups with low socioeconomic status or poor literacy. The data show that presenting complex information in simplified formats improves patients' satisfaction, comprehension, and decision making and leads to improved behavioural outcomes (eg, adherence to prescription regimens).^14^ During the development of this leaflet we were also mindful of guidelines on informed choice.^22^ We had previously done structured interviews to identify areas of the standard information booklet susceptible to being misunderstood and used this information to design the gist leaflet. The effects of the leaflet on comprehension were assessed in cognitive interviews and a trial in primary care, which showed improved comprehension compared with the standard information.^19^, ^20^

### Trial 2: narrative leaflet

Narrative information is recognised as an effective communication aid for individuals with poor literacy.^23^ The narrative leaflet (appendix) was created on the basis of information obtained in interviews with people who had participated in the BCSP. Material was selected to reflect screening outcomes: a normal gFOBT result, polyp removal, and screen-detected colorectal cancer. We tested the efficacy of the leaflet in a trial in primary care, in which respondents reported being more inclined to take part in screening than with standard information.^13^

### Trial 3: general practice endorsement

International evidence shows that screening invitations sent by individuals' family doctors improve uptake in groups with low socioeconomic status.^11^, ^14^, ^15^, ^16^ BCSP invitation letters are sent from the hubs, but there is space on the letter to mention the support of the general practice with which an individual is registered, although not the named general practitioner. We created a general practice endorsement that appeared as a banner across the invitation letter (appendix). We sought consent from all practices in England (n=8142), in collaboration with a primary care advisory group and HSCIC, by sending each one a written invitation to be part of the trial, followed up by reminders 4 and 8 weeks later. Permission to link the practice address to the invitation was granted by 6480 (80%) of practices.

### Trial 4: enhanced reminder letter

Reminders have a slight impact on uptake, including in very-low-income groups.^13^, ^15^ They also provide an opportunity to restate the screening offer. We created an enhanced reminder letter that was aimed specifically at individuals who had not responded to the initial invitation. A simple restatement of the screening offer was made in a short paragraph added to the end of the standard letter, and a banner was added to the start of the letter that said “A reminder to you” (appendix). The offer restatement text had been refined through a series of focus groups and a review of reminder-related queries made by patients to the BCSP's telephone helpline.

### Randomisation

Randomisation was based on day of invitation, with “day within hub” constituting the randomisation unit. Trials 1 and 2 were run over 10 consecutive days in November, 2012, and March, 2013, respectively. Trials 3 and 4 were run over 20 consecutive days in June to July, 2013, and July to August, 2013, respectively. 2 weeks before the start of each intervention, a random number sequence was generated with a continuous random number for each hub day, and numbers higher than the median were allocated to the intervention groups and those lower than the median to the control groups. For trials 1 and 2, randomisation schedules were created and sent to a printing company (Real Digital International, Croydon, UK), for the Southern, London, and Eastern hubs, and to the in-house invitation service for the North East and Midlands and North West hubs. For trials 3 and 4, randomisation was done through the Bowel Cancer Screening System, which identifies the eligible population for screening in each hub. For trial 3 this system was modified to enable selection of invitees who belonged to general practices that had agreed to endorse the BCSP before the creation of the invitation letters. Although masking of hubs was not possible, there was no direct contact between hub staff sending the invitations or reminders and invitees. Each study group was unaware of the materials received by the other study groups unless members of the same household received different invitation types or a person had been invited previously.

### Outcome measures

The primary outcome was socioeconomic status gradient in screening uptake over quintiles of Index of Multiple Deprivation (IMD). We defined screening uptake as the proportion of routinely invited individuals that returned gFOBT kits within 18 weeks of being sent invitations that led to an result of normal or abnormal (with clinical referral for prosepctive colonoscopy) by the date of data extraction (18 weeks after the last day of the intervention). We used the English IMD 2010 score associated with home postcodes to classify socioeconomic status.^24^ IMD is an area-based measure that combines income, employment, health and disability, education, skills and training, barriers to housing and services, crime, and living environment, into a deprivation score. The scores are assigned to small geographical areas termed lower-layer super output areas, of which there are 32 844 in England, each covering about 1500 individuals. Each recipient's postcode was linked to the relevant lower-layer super output area. The IMD scores were grouped into quintiles based on national distributions with use of predefined national cutoffs.

The age and sex of each recipient were obtained from the BCSP database. We gathered information on whether each individual was being invited for the first time (prevalent first-time episode), being sent a biennial invitation having previously not responded (prevalent episode), or being sent a biennial invitation having been screened before (incident episode).

Secondary outcomes were the median number of days to return the gFOBT kit and the proportion of spoilt and undelivered test kits, by intervention and IMD quintile. We also assessed the marginal cost per additional person receiving each intervention, calculated with actual costs incurred during the study and valued according to market prices.

### Statistical analysis

Target sample sizes for each trial were estimated to detect an average increase in uptake of 3 percentage points, based on 1 percentage point increase in the least deprived quintile and 5 percentage points in the most deprived quintile, with 90% power and p<0·05. The final calculation was based on the demographic composition of the hub that required the largest sample size (Midlands and North West). Because invitations were randomised by day, but the number of invitations sent per day varies, we applied an inflation factor of 1·7 to ensure that the sample size would confer adequate statistical power. We calculated, therefore, that 46 000 individuals (23 000 per group) would be needed for each of trials 1 and 2. However, due to the volume of invitations sent out each week, this target would have been achieved within 5 days of invitations, and because small numbers of clusters increase the risk of bias^25^ we had specified 10-day intervention periods. The final sample size for each of these trials, therefore, was 140 000–160 000 individuals. The estimated sample size for trial 3 (84 000) assumed agreement from 30% of practices and, therefore, the required sample size was increased by a factor of 100/30 to a target of 280 000. To achieve this sample size would need 14 days of sending invitations, but we had allowed 20 days. The target sample size for trial 4 was 140 000, which reflected the fact that the daily number of reminders is substantially lower than first invitations.

The intervention periods for trials 3 and 4 overlapped because of initial interest in using a factorial design to investigate the combined effect of adding the general practice endorsement to the enhanced reminder letter as well as the initial invitation. Owing to space constraints on the reminder letter, however, we could not proceed with this plan, but it meant that some individuals in trial 3 who did not respond to their invitation within 28 days were included in trial 4.

The primary outcome was analysed by logistic regression. Odds ratios (ORs), p values, and 95% CIs were calculated with conservative variance estimation to allow for the potential clustering effects, and were controlled for hub, age, sex, and screening round.^25^, ^26^ The conservative variance analysis allowed for correlation of individuals within randomisation clusters but not between clusters, and used the Huber-White information sandwich method to estimate variance.^27^, ^28^ The primary outcome was tested by the two-factor interaction term between intervention group and IMD quintile. Analyses were done on an intention-to-treat basis. Analyses were done with SAS version 9.3 and Stata version 12.1. This study is registered, number ISRCTN74121020.

### Role of the funding source

The funder of the study had no role in study design, data collection, data analysis, data interpretation, or writing of the report. The corresponding author had full access to all the data in the study and had final responsibility for the decision to submit for publication.

## Results

Baseline characteristics were well balanced for each trial and showed that the populations were representative of that served by the BCSP (table 1).^6^ Overall uptake per study was 57·4%, 57·7%, 57·9%, and 25·4% in trials 1, 2, 3, and 4, respectively; the proportion is low in trial 4 because it only targeted individuals who had not responded within 4 weeks of the invitation letter. In all trials, uptake was strongly and negatively associated with deprivation, with the difference between the least and most deprived quintiles in each control arm ranging from 20 to 24 percentage points (table 2).

In trial 1, uptake was similar in the intervention and control groups and the least and the most deprived quintiles (table 2). The socioeconomic status gradient in screening uptake did not differ by IMD quintile and no significant increase was seen in overall uptake (table 3, appendix). The median number of days to return the test kit was 23 (range 12–126) in the intervention group and 22 (11–126) in the control group. Median response times did not differ by IMD quintile (appendix). The proportions of spoilt and undelivered test kits were very small and were similar in the two groups and across IMD quintiles (appendix).

For trial 2, uptake did not differ between groups or between least and most deprived quintiles (table 2). The socioeconomic status gradient in screening uptake did not differ across deprivation quintiles and no effect was seen on overall uptake (table 3). The median number of days to return the test kit was 26 (range 11–126) in the intervention group and 26 (10–126) in the control group, and timing did not differ by IMD quintile (appendix). The proportions of spoilt and undelivered test kits were similar in the two study groups and across IMD quintiles (appendix).

In trial 3, general practice endorsement was associated with a slight percentage point differential in uptake between the least and most deprived quintiles (table 2). We also noted a slight gradient in uptake related to socioeconomic status, but this effect was not significant (table 3). Although the unadjusted OR indicated little effect on overall uptake (appendix), the effect became significant after adjustment (table 3). This change is mainly due to differences in effect sizes between study groups for screening episode (table 2). The median number of days taken to return the test kit was 22 (range 8–126) for the intervention group and 23 (11–126) for the control group, and timings were similar across IMD quintiles (appendix). The proportions of spoilt and undelivered test kits were similar in the two study groups and across IMD quintiles (appendix).

In trial 4, the enhanced reminder letter was associated with a difference in uptake between the intervention and control groups and the lowest and highest deprivation quintiles (table 2). We found a significant interaction between uptake and IMD quintile, with a stronger effect seen in the most deprived than in the least deprived quintile (table 3). The unadjusted OR for overall uptake did not differ between study groups, but the effect became significant after adjustment (table 3). The median number of days to return test kits was 11 (range −4 to 89) in the intervention group and 11 (0–89) in the control group, and did not differ across IMD quintiles (appendix). The proportions of spoilt and undelivered test kits also did not differ (appendix).

Owing to the overlap in timing, the inclusion and randomisation statuses of trials 3 and 4 have been cross-tabulated (appendix). A larger proportion of recipients in the trial 3 intervention group was randomised to trial 4 than in the control group (49·4% *vs* 44·6%). Nevertheless, the unadjusted OR for participation within 4 weeks associated with general practice endorsement (before the reminder could have been received) was 1·06 (95% CI 0·99–1·04), which was higher than that for overall uptake (1·03, 0·95–1·11; appendix). Similarly, the unadjusted OR associated with the enhanced reminder letter for recipients who were not enrolled in trial 3 was 1·06 (95% CI 0·93–1·21), which is also higher than the unadjusted OR for overall uptake (1·04, 0·95–1·14; appendix). Furthermore, the OR associated with receiving the enhanced reminder letter adjusted for trial 3 status was 1·04 (95% CI 0·95–1·14), which matched the unadjusted OR.

The average marginal costs of providing the gist and narrative leaflets were, respectively, £0·04 and £0·05 per person screened. For the general practice endorsement and enhanced reminder letters, a one-off cost of £78 000 was incurred to modify both in the BCSP IT system. As this cost would not be incurred again if the interventions were implemented, there was no marginal cost per person screened.

## Discussion

Reducing socioeconomic inequalities in cancer mortality is a priority worldwide. Cancer screening is a major component of efforts to bring forward diagnosis to earlier, more treatable stages. Even in the UK, where screening incurs no financial cost to the individual, uptake declines with increasing socioeconomic deprivation.^4^, ^5^, ^6^ Our four trials enabled assessment of interventions designed to lessen inequalities in uptake in large study populations. An important strength of ASCEND was that the trials were powered to measure the effects of interventions in relation to socioeconomic status in the total eligible population, rather than merely focusing on disadvantaged groups. The interventions, therefore, had the potential to reach a large number of people who had not previously participated in the screening programme. Use of routinely collected data enabled us to include most of the potential study population in our analysis, except for a very small group of people without IMD scores for their postcodes. Each intervention was also based on a well established rationale and empirical data, and was developed through a structured, comprehensive process. Only the enhanced reminder letter, however, led to a reduction in the socioeconomic status gradient in uptake. The gist and narrative leaflet interventions had no effect on uptake. The general practice endorsed letter was associated with increased uptake overall, but did not modify the socioeconomic status gradient. None of our interventions promoted early response or a reduced number of spoilt test kits. The numbers of undelivered information packs also did not differ, by group or IMD quintile.

The gist and narrative leaflets in trials 1 and 2 were designed to make the offer of screening more visible to people with poor literacy skills. Both leaflets showed this potential when their effects were assessed on the basis of knowledge, attitudes, or intention to participate in screening.^16^, ^20^ A possible explanation for lack of effect in these trials is that the determinants of intention can differ from the determinants of action, and that the leaflets only affected the former. Another possible explanation relates to the fact that the two leaflets had to be added to the existing invitation or information rather than being provided as an alternative. Consequently, although the leaflets were designed to be simple, they increased the total mass of written material and might have undermined the goal of making the screening offer more visible.

The general practice endorsement significantly increased overall uptake, but the effect size was smaller than in many previous studies.^10^, ^11^, ^12^, ^13^ This difference was probably due to previous studies mostly using letters sent directly from the general practitioner or with the individual doctors' signatures on the letters. We were unable to apply such alterations for logistical reasons, which might have diluted the efficacy of this intervention.

Owing to general practice endorsement having previously shown effects in low-income groups,^13^ we had hypothesised that the effect in this study would have been stronger in lower than in higher socioeconomic groups. Previous studies, however, had not been powered to assess effects on the socioeconomic gradient. The large size of the ASCEND trial, though, means that our negative result is definitive, at least with the format of endorsement that we used. Nevertheless, in view of the high level of agreement by practices to endorse the screening programme and the absence of a marginal cost per person screened, we recommend that the BCSP considers adding the general practice endorsement banner to screening invitation letters.

One intervention that reduced the socioeconomic gradient was the enhanced reminder letter, which also slightly increased overall uptake. The aim of this intervention was to offer anyone who had not engaged with the original materials an additional opportunity to see and consider the screening offer. Unlike the gist and narrative leaflets, this enhancement was incorporated into the one-page reminder letter and, therefore, might have had higher visibility. Although the change in the gradient was small (as was the effect on overall uptake), this intervention was also virtually cost-free and, therefore, offers a practical way for the screening programme to reduce the socioeconomic gradient in uptake. As this addition to the reminder letter was minor, investigating the cost-effectiveness of adding a second reminder or using alternative channels, such as text messaging, to reiterate the offer of screening could be worthwhile.

ASCEND had some limitations. People from deprived backgrounds are likely to be struggling with multiple social and economic challenges, making it difficult for them to prioritise cancer screening. These upstream issues, however, cannot be addressed by minor variations in the format of a screening offer. Nonetheless, ensuring that the screening offer is not only mailed to all eligible adults but is also appropriate for a wide range of levels of literacy should be a goal of NHS screening programmes. We did not address broader attitudes to cancer. For example, cancer fatalism and other negative attitudes are more prevalent in groups with low than with high socioeconomic status, and fatalism has been associated with delayed diagnosis.^29^ Negative attitudes are not easily modified with simple written materials. We did not address various other downstream barriers, of which the most well established is the unpleasantness associated with completing the test kit. If the BCSP implements the faecal immunochemical test for haemoglobin, which typically only requires one stool sample, inequalities in participation might be reduced.^30^, ^31^

The sampling timeframe for trials 3 and 4 overlapped because the original plan had been that individuals randomised to receive the general practice endorsement letter in trial 3 would have a similar banner on their reminder letters in trial 4. This approach, however, was logistically impossible because of space limitations on the page. Thus, we analysed the two trials separately but did supplementary analyses to test whether the overlap had resulted in overestimation of effects for either intervention. We found no evidence of bias and, therefore, conclude that the overlap, although not desirable, did not compromise our results.

The inclusion of strategies in routine programme delivery provides a model for future research, and there might be scope to test changes to the interventions that could strengthen the effects. For instance, supplying all the necessary screening information in smaller instalments by integrating additional communication points into the screening pathway might improve the visibility and efficacy of the gist and narrative leaflets. The use of additional reminder letters might, through a process of elimination, help to target the most deprived populations.

In conclusion, the enhanced reminder letter was the only strategy to significantly reduce the socioeconomic gradient, and overall uptake was only increased by this and the general practice endorsement intervention. In view of the very low expense, these interventions could be implemented with minimum cost or disruption to the existing programme. Our findings suggest that tailoring of information delivery to the communities being served might be useful. A possibility in poor-literacy groups is to supplement mailed information with direct contact with health professionals. The results of our four trials illustrate the difficulty of addressing inequality in screening uptake within an organised programme, but highlight the importance of continuing to investigate new strategies.

## Figures and Tables

**Table 1:** Baseline characteristics of trial population

		**Trial 1**		**Trial 2**		**Trial 3**		**Trial 4**	
		Standard information and gist leaflet (n=84 283)	Standard information (n=78 791)	Standard information and narrative leaflet (n=73 450)	Standard information (n=76 421)	GP endorsed invitation letter (n=130 876)	Standard letter (n=133 449)	Enhanced reminder letter (n=77 739)	Standard letter (n=90 002)
Age (years)		66 (59–74)	66 (59–74)	65 (59–74)	65 (59–74)	65 (59–74)	65 (59– 74)	65 (59–74)	64 (59–74)
Sex									
	Female	43 195 (51·2%)	40 671 (51·4%)	37 937 (51·5%)	39 086 (51·0%)	66 986 (51·0%)	68 591 (51·2%)	37 747 (48·4%)	43 574 (48·2%)
	Male	41 226 (48·8%)	38 433 (48·6%)	35 785 (48·5%)	37 609 (49·0%)	64 437 (49·0%)	65 420 (48·8%)	40 320 (51·6%)	46 839 (51·8%)
Screening episode									
	Prevalent first time	13 034 (15·4%)	12 410 (15·7%)	15 281 (20·7%)	12 510 (16·3%)	22 287 (17·0%)	23 582 (17·6%)	14 483 (18·6%)	21 271 (23·5%)
	Prevalent	26 368 (31·2%)	24 551 (31·0%)	22 209 (30·1%)	22 892 (29·8%)	40 441 (30·8%)	40 295 (30·1%)	39 862 (51·1%)	43 329 (47·9%)
	Incident	45 019 (53·3%)	42 143 (53·3%)	36 232 (49·1%)	41 293 (53·8%)	68 695 (52·3%)	70 134 (52·3%)	23 722 (30·4%)	25 813 (28·6%)
Programme administrative hub									
	1	22 469 (26·6%)	24 369 (30·8%)	21 421 (29·1%)	21 118 (27·5%)	35 993 (27·4%)	34 598 (25·8%)	22 051 (28·2%)	25 490 (28·2%)
	2	20 651 (24·5%)	21 004 (26·6%)	20 667 (28·0%)	16 723 (21·8%)	31 760 (24·2%)	40 550 (30·3%)	19 131 (24·5%)	23 107 (25·6%)
	3	7416 (8·8%)	6636 (8·4%)	8509 (11·5%)	8795 (11·5%)	11 818 (9·0%)	13 255 (9·9%)	10 809 (13·8%)	10 385 (11·5%)
	4	13 614 (16·1%)	12 858 (16·3%)	13 053 (17·7%)	12 900 (16·8%)	21 272 (16·2%)	21 439 (16·0%)	12 291 (15·7%)	12 796 (14·2%)
	5	20 271 (24·0%)	14 237 (18·0%)	10 072 (13·7%)	17 159 (22·4%)	30 580 (23·3%)	24 169 (18·0%)	13 785 (17·7%)	18 635 (20·6%)
IMD quintile*									
	1 (least deprived)	19 055 (22·6%)	18 554 (23·5%)	17 027 (23·2%)	17 073 (22·3%)	30 350 (23·1%)	31 381 (23·4%)	15 933 (20·4%)	18 928 (20·9%)
	2	19 787 (23·5%)	18 295 (23·2%)	16 517 (22·5%)	17 675 (23·1%)	30 952 (23·6%)	31 340 (23·4%)	16 594 (21·3%)	19 446 (21·5%)
	3	18 320 (21·7%)	15 993 (20·3%)	15 287 (20·8%)	16 161 (21·1%)	27 950 (21·3%)	28 181 (21·0%)	16 092 (20·6%)	18 286 (20·2%)
	4	14 747 (17·5%)	13 469 (17·1%)	12 897 (17·6%)	13 385 (17·5%)	22 450 (17·1%)	23 007 (17·2%)	14 679 (18·8%)	16 853 (18·6%)
	5 (most deprived)	12 374 (14·7%)	12 660 (16·0%)	11 722 (16·0%)	12 127 (15·9%)	19 174 (14·6%)	19 540 (14·6%)	14 441 (18·5%)	16 489 (18·2%)
	Score missing	138	133	272	274	547	562	328	411

Data are median (range) or number (%). GP=general practice. IMD=Index of Multiple Deprivation.

* Quintile categories were based on national cutoffs.^26^

**Table 2:** Proportions of people who took up screening

		**Number (%) in trial 1**		**Number (%) in trial 2**		**Number (%) in trial 3**		**Number (%) in trial 4**	
		Standard information and gist leaflet (n=84 283)	Standard information (n=78 791)	Standard information and narrative leaflet (n=73 450)	Standard information (n=76 421)	GP endorsed invitation letter (n=130 876)	Standard letter (n=133 449)	Enhanced reminder letter (n=77 739)	Standard letter (n=90 002)
Adequately screened (overall)		48 653 (57·6%)	45 290 (57·3%)	41 822 (56·7%)	44 904 (58·5%)	76 520 (58·2%)	77 122 (57·5%)	20 166 (25·8%)	22 712 (25·1%)
Age (years)									
	60–64	19 727 (54·9%)	18 200 (54·2%)	18 264 (53·3%)	19 014 (55·2%)	33 331 (55·9%)	33 480 (54·8%)	10 251 (26·7%)	12 229 (26·1%)
	65–69	18 657 (60·8%)	17 346 (61·1%)	14 673 (60·9%)	16 673 (62·4%)	27 382 (61·0%)	27 466 (60·5%)	6674 (26·8%)	6898 (24·8%)
	70–74	10 269 (57·7%)	9744 (56·9%)	8885 (57·9%)	9217 (59·2%)	15 807 (58·7%)	16 176 (58·8%)	3241 (21·9%)	3585 (22·6%)
Sex									
	Female	25 585 (59·2%)	24 017 (59·1%)	22 499 (59·3%)	23 811 (60·9%)	40 707 (60·8%)	41 290 (60·2%)	10 267 (27·2%)	11 511 (26·4%)
	Male	23 068 (56·0%)	21 273 (55·4%)	19 323 (54·0%)	21 093 (56·1%)	35 813 (55·6%)	35 832 (54·8%)	9899 (24·5%)	11 201 (23·9%)
Screening episode									
	Prevalent first time	6466 (49·6%)	5981 (48·2%)	7678 (50·2%)	6231 (49·8%)	11 465 (51·4%)	11 646 (49·4%)	3739 (25·8%)	5398 (25·4%)
	Prevalent	3836 (14·5%)	3479 (14·2%)	3113 (14·0%)	3284 (14·3%)	5675 (14·0%)	5357 (13·3%)	2394 (6·0%)	2329 (5·4%)
	Incident	38 351 (85·2%)	35 830 (85·0%)	31 031 (85·6%)	35 389 (85·7%)	59 380 (86·4%)	60 119 (85·7%)	14 033 (59·2%)	14 985 (58·1%)
IMD quintile*									
	1 (least deprived)	12 547 (65·8%)	12 178 (65·6%)	11 005 (64·6%)	11 411 (66·8%)	19 792 (65·2%)	20 716 (66·0%)	5522 (34·7%)	6601 (34·9%)
	2	12 305 (62·2%)	11 412 (62·4%)	10 253 (62·1%)	11 080 (62·7%)	19 530 (63·1%)	19 604 (62·6%)	5107 (30·8%)	5782 (29·7%)
	3	10 732 (58·6%)	9335 (58·4%)	8911 (58·3%)	9601 (59·4%)	16 571 (59·3%)	16 336 (58·0%)	4316 (26·8%)	4578 (25·0%)
	4	7663 (52·0%)	6987 (51·9%)	6535 (50·7%)	7083 (52·9%)	11 902 (53·0%)	11 839 (51·5%)	3104 (21·1%)	3436 (20·4%)
	5 (most deprived)	5322 (43·0%)	5316 (42·0%)	4966 (42·4%)	5580 (46·0%)	8433 (44·0%)	8324 (42·6%)	2040 (14·1%)	2198 (13·3%)
	Missing IMD score	84	62	152	149	292	303	77	117

GP=general practice. IMD=Index of Multiple Deprivation.

* Quintile categories were based on national cutoffs.^26^

**Table 3:** Adjusted odds for screening uptake, overall and by deprivation quintile

	**Trial 1 (standard information and gist leaflet)**	**Trial 2 (standard information and narrative leaflet)**	**Trial 3 (GP endorsed invitation letter)**	**Trial 4 (enhanced reminder letter)**
Overall uptake	1·03 (0·99–1·06) p=0·15	1·00 (0·96–1·03) p=0·80	1·07 (1·04–1·10) p<0·0001	1·07 (1·03–1·11) p=0·001
1 (least deprived)	1·06 (1·01–1·11)	0·98 (0·93–1·04)	1·04 (0·99–1·08)	1·00 (0·94–1·06)
2	1·02 (0·97–1·07)	1·00 (0·94–1·06)	1·06 (1·02–1·10)	1·04 (0·98–1·11)
3	1·00 (0·94–1·08)	1·05 (0·97–1·13)	1·08 (1·03–1·13)	1·13 (1·06–1·20)
4	1·01 (0·94–1·08)	1·00 (0·94–1·06)	1·09 (1·04–1·15)	1·09 (1·02–1·17)
5 (most deprived)	1·04 (0·96–1·12) p_interaction_=0·68	0·92 (0·86–0·98) p_interaction_=0·11	1·07 (1·01–1·13) p_interaction_=0·49	1·11 (1·04–1·20) p_interaction_=0·005

Data are odds ratios (95% CI). *Adjusted for hub, age, sex, and screening episode.
