# Supplementary material for: Effects of evidence-based strategies to reduce the socioeconomic gradient of uptake in the English NHS Bowel Cancer Screening Programme (ASCEND): four cluster-randomised controlled trials
Source: Lancet. 2016 Feb 20;387(10020):751–9. doi: 10.1016/S0140-6736(15)01154-X (PMC4761689; doi:10.1016/S0140-6736(15)01154-X)
Supplement: Supplementary appendix [file mmc1.pdf]

THE LANCET

Supplementary appendix

This appendix formed part of the original submission and has been peer reviewed. We post it as supplied by the authors.

Supplement to: Wardle J, von Wagner C, Kralj-Hans I, et al. Effects of evidence-based strategies to reduce the socioeconomic gradient of uptake in the English NHS Bowel Cancer Screening Programme (ASCEND): four cluster-randomised controlled trials. *Lancet* 2015; published online Dec 8. http://dx.doi.org/10.1016/S0140-6736(15)01154-X.

APPENDIX

Gist leaflet (trial 1)

ASC GIST/11/12

NHS Bowel Cancer Screening Programme: The Essentials

- Bowel cancer is the third most common cancer
- The FOB test can find hidden signs of bowel cancer early
- Doing the FOB test every 2 years lowers the risk of dying from bowel cancer
- Men and women aged 60-74 are sent the FOB test to do at home

Developed by University College London with funding from the National Institute for Health Research's programme grants for applied health research

NHS National Institute for Health Research

Would you like to know more? See inside for details

How does the FOB test work?

- The FOB (Faecal Occult Blood) test checks for tiny amounts of blood in stools (poo) that might not be seen by the eye
- Blood in stools can be a sign of bowel cancer
- The FOB test kit is sent to your home
- The FOB test is easy to do
- You do the FOB test at home by putting small amounts of stool onto a test kit
- You send the test kit back to the laboratory in a special freepost envelope

What happens after you've done the FOB test?

- You get your FOB result through the post within 2 weeks
- Most people (98 out of 100) get a normal result
- If you have a normal result you will be sent another FOB test every 2 years up to age 74
- A small number of people (2 out of 100) get an abnormal result
- If you get an abnormal result, you will get an appointment to talk about further testing
- For most people, the follow-up test will show there is no bowel cancer
- If bowel cancer is found, it is likely to be at an early stage when treatment is more successful

How accurate is the FOB test?

- Doing the FOB test every 2 years lowers the risk of dying from bowel cancer
- Like all screening tests, the FOB test is not 100% accurate
- Bowel cancer can be missed if there is no bleeding at the time the FOB test is done

Where can I get more information?

For more information see the enclosed leaflet:

'Bowel Cancer Screening: The Facts'

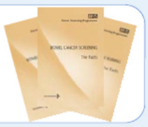

Narrative leaflet (trial 2)

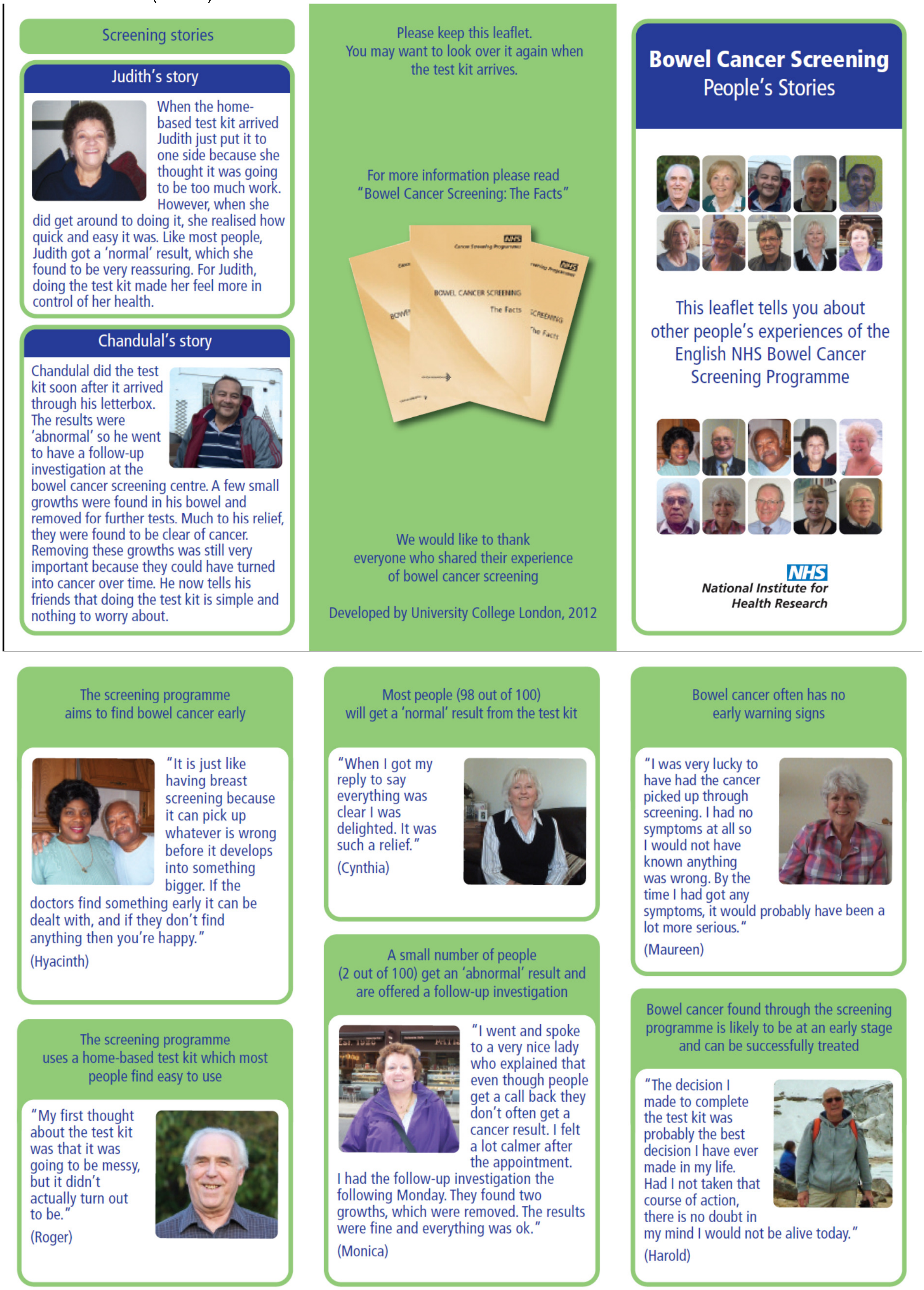

Screening stories

Judith's story

When the home-based test kit arrived Judith just put it to one side because she thought it was going to be too much work. However, when she did get around to doing it, she realised how quick and easy it was. Like most people, Judith got a 'normal' result, which she found to be very reassuring. For Judith, doing the test kit made her feel more in control of her health.

Chandulal's story

Chandulal did the test kit soon after it arrived through his letterbox. The results were 'abnormal' so he went to have a follow-up investigation at the bowel cancer screening centre. A few small growths were found in his bowel and removed for further tests. Much to his relief, they were found to be clear of cancer. Removing these growths was still very important because they could have turned into cancer over time. He now tells his friends that doing the test kit is simple and nothing to worry about.

Please keep this leaflet.
You may want to look over it again when the test kit arrives.

For more information please read "Bowel Cancer Screening: The Facts"

We would like to thank everyone who shared their experience of bowel cancer screening

Developed by University College London, 2012

Bowel Cancer Screening
People's Stories

This leaflet tells you about other people's experiences of the English NHS Bowel Cancer Screening Programme

NHS
National Institute for Health Research

The screening programme aims to find bowel cancer early

"It is just like having breast screening because it can pick up whatever is wrong before it develops into something bigger. If the doctors find something early it can be dealt with, and if they don't find anything then you're happy."
(Hyacinth)

The screening programme uses a home-based test kit which most people find easy to use

"My first thought about the test kit was that it was going to be messy, but it didn't actually turn out to be."
(Roger)

Most people (98 out of 100) will get a 'normal' result from the test kit

"When I got my reply to say everything was clear I was delighted. It was such a relief."
(Cynthia)

A small number of people (2 out of 100) get an 'abnormal' result and are offered a follow-up investigation

"I went and spoke to a very nice lady who explained that even though people get a call back they don't often get a cancer result. I felt a lot calmer after the appointment. I had the follow-up investigation the following Monday. They found two growths, which were removed. The results were fine and everything was ok."
(Monica)

Bowel cancer often has no early warning signs

"I was very lucky to have had the cancer picked up through screening. I had no symptoms at all so I would not have known anything was wrong. By the time I had got any symptoms, it would probably have been a lot more serious."
(Maureen)

Bowel cancer found through the screening programme is likely to be at an early stage and can be successfully treated

"The decision I made to complete the test kit was probably the best decision I have ever made in my life. Had I not taken that course of action, there is no doubt in my mind I would not be alive today."
(Harold)

GP endorsement letter (trial 3)

NHS No: 123 456 7890

Bowel Cancer Screening Programme

Midlands and North West Bowel Cancer Screening Programme Hub
Rugby Hospital
Rugby
The Midlands
RG3 2TH

Freephone Helpline: 0800 707 60 60
Email: bowel.screening@uhcw.nhs.uk

Open: Mon to Fri 9:00am to 5:00pm
Sat 9:00am to 12:00pm
Sun 10:30am to 12:30pm

10 June 2013

Mrs A B Example
1 The Street
Newtown
AB1 1CD

S1# 4301/1/264

Dear Mrs A B Example

Your GP practice, WWWW WWWW WWWW WWWW WWWW WWWW WWWW WWWWW, supports the Bowel Cancer Screening Programme

This is an invitation to take part, or take part again, in the NHS Bowel Cancer Screening Programme. The programme aims to detect bowel cancer early, when successful treatment and cure is more likely. Screening is offered every two years to people aged 60-69 who are registered with a GP in England. We are starting to extend the screening age range, so if you are aged 70-74, you are being invited as part of this process.

You will be sent a test kit with full instructions in about two weeks. The kit is simple to use in the privacy of your own home. If you want to be screened, wait until the kit arrives, follow the instructions, and return the kit in the **reply paid** envelope provided. You will get your results by letter within 2 weeks.

We do not have your medical history, and screening is not appropriate for everyone. **If you have already been referred to hospital by your GP for bowel investigations, or if you have had previous bowel surgery, then screening may not be appropriate for you. Please call us for advice.** If you don't wish to be screened, then please call and let us know. The **Freephone** number for all calls is at the top of this letter (calls are free from UK landlines).

If you need help from family or a carer in order to use the kit, please call us (or ask them to call us) for further important information. You can also use the **Freephone** number if you have any questions about taking part in the programme. Finally, please take the time to read the enclosed leaflet 'Bowel Cancer Screening - The Facts', which may help to answer any questions you may have.

Yours sincerely

CR329 Local Signatory

SAMPLE

Enhanced reminder letter (trial 4)

NHS No: 123 456 7890

Bowel Cancer Screening Programme

Midlands and North West Bowel Cancer Screening Programme Hub
Rugby Hospital
Rugby
The Midlands
RG3 2TH

Freephone Helpline: 0800 707 60 60
Email: bowel.screening@uhcw.nhs.uk

Open: Mon to Fri 9:00am to 5:00pm
Sat 9:00am to 12:00pm
Sun 10:30am to 12:30pm

10 July 2013

Mr A B Example
1 The Avenue
Oldtown
ZY9 9XW

S10# 1108/13/205

Dear Mr A B Example

A REMINDER TO YOU

You were recently sent a test kit from the NHS Bowel Cancer Screening Programme. This is a simple test you can carry out at home, which checks for signs of abnormalities (such as polyps or cancers) in the bowel. The test is designed to detect tiny traces of blood (not visible to the naked eye) in bowel motions.

If blood is found, then a further examination called a colonoscopy is recommended. This examination looks at the inside of the bowel. Only around 2 in every 100 people completing the home test kit are advised to have a colonoscopy.

We do not appear to have received your completed test kit. If you returned your kit **more than 7 days ago** but have not received a result, please call the **Freephone** number at the top of this letter. (If you returned your kit within the last 7 days please ignore this letter).

If you have any queries or concerns about using the kit, would like a replacement kit, or do not wish to take part in the screening programme, please contact us on the **Freephone** number.

If we do not hear from you within 13 weeks, you will be discharged from this screening round. You will be invited to participate in screening again in 2 years time, unless you have reached your 75th birthday by then. People aged 75 or over are not automatically invited for screening, but can request a test kit by calling the **Freephone** number above.

Doing the test kit is important because the risk of bowel cancer increases as you get older. If bowel cancer is found early, treatment is more successful. It's never too late to do the test. Call Freephone 0800 707 60 60 if you need to speak to a helpline assistant.

Yours sincerely

CR225 Local Signatory

SAMPLE

Unadjusted odds ratios (ORs)* and 95% confidence intervals for screening uptake by IMD quintile, in each trial, with conservative variance.

IMD quintile	Trial 1	Trial 2	Trial 3	Trial 4
	Standard + gist	Standard + narrative	Standard with GP endorsement	Standard with enhanced reminder
Overall	1.02 (0.92-1.13) p=0.77	0.93 (0.81-1.06) p=0.27	1.03 (0.95-1.11) p=0.49	1.04 (0.95-1.14) p=0.42
1 (Least deprived)	1.01 (0.92-1.11)	0.91 (0.81-1.01)	0.97 (0.88-1.05)	0.99 (0.90-1.09)
2	0.99 (0.91-1.09)	0.97 (0.88-1.08)	1.02 (0.95-1.10)	1.05 (0.97-1.14)
3	1.01 (0.90-1.13)	0.95 (0.85-1.08)	1.06 (0.98-1.14)	1.10 (1.02-1.18)
4	1.00 (0.91-1.11)	0.91 (0.78-1.06)	1.06 (0.98-1.16)	1.05 (0.96-1.14)
5 (Most deprived)*	1.04 (0.92-1.18) p = 0.61	0.86 (0.74-1.00) p = 0.44	1.06 (0.97-1.15) p = 0.27	1.07 (0.97-1.18) p = 0.28

GP=general practice. *p-values of the SES (IMD quintile) x group interaction.

Gist Intervention: Time taken to return the gFOBt kit by IMD quintile.

Variable	Trial 1	Standard information
Time taken to return the gFOBt kit in days:	median (range) 23 (12-126)	median (range) 22 (11-126)
1st quintile (least deprived)	24 (12-126)	22 (12-126)
2nd quintile	23 (12-126)	22 (12-126)
3rd quintile	23 (12-126)	22 (12-126)
4th quintile	23 (13-126)	21 (11-126)
5th quintile (most deprived)	21 (12-126)	21 (12-126)

Narrative Intervention: Time taken to return the gFOBt kit by IMD quintile.

Variable	Trial 2	Standard information
Time taken to return the gFOBt kit in days:	median (range) 26 (11-126)	median (range) 26 (10-126)
1st quintile (least deprived)	26 (13-126)	27 (13-126)
2nd quintile	26 (11-126)	27 (13-125)
3rd quintile	25 (11-126)	26 (12-125)
4th quintile	25 (13-125)	26 (10-126)
5th quintile (most deprived)	24 (13-126)	26 (12-126)

GPE Intervention: Time taken to return the gFOBt kit by IMD quintile.

Variable	Trial 3	Standard information
	median (range)	median (range)
Time taken to return the gFOBt kit in days:	22 (8-126)	23 (11-126)
1st quintile (least deprived)	23 (12-126)	24 (11-126)
2nd quintile	23 (12-126)	24 (13-126)
3rd quintile	22 (11-126)	23 (11-126)
4th quintile	22 (8-126)	22 (13-126)
5th quintile (most deprived)	21 (12-126)	22 (12-125)

Enhanced Reminder Intervention: Time taken to return the gFOBt kit by IMD quintile.

Variable	Trial 4	Standard information
	median (range)	median (range)
Time taken to return the gFOBt kit in days:	11 (-4-89)	11 (0-89)
1st quintile (least deprived)	11 (-4-89)	11 (0-89)
2nd quintile	11 (-4-89)	11 (0-89)
3rd quintile	11 (-4-89)	11(0-89)
4th quintile	11 (-4-89)	11 (0-89)
5th quintile (most deprived)	11(-2-89)	11 (0-89)

Proportion of people with spoilt kits and IMD quintile.

	Trial 1 (N=163,525)		Trial 2 (N=150,417)		Trial 3 (N=265,434)		Trial 4 (N=168,480)	
	Standard + Gist	Standard	Standard+ Narrative	Standard	Standard with GP endorsement	Standard	Standard with Enhanced Reminder	Standard
	% (n)	% (n)	% (n)	% (n)	% (n)	% (n)	% (n)	% (n)
**IMD quintiles**								
1 (least deprived)	0.6 (122)	0.6 (118)	0.6 (103)	0.5 (90)	0.6 (189)	0.6 (185)	0.8 (128)	0.8 (156)
2	0.6 (126)	0.6 (113)	0.7 (109)	0.6 (99)	0.6 (200)	0.7 (204)	0.8 (130)	0.8 (154)
3	0.8 (141)	0.7 (104)	0.8 (122)	0.7 (118)	0.7 (199)	0.7 (202)	0.9(151)	0.8 (141)
4	0.9 (139)	0.9 (122)	1.1 (143)	1.0 (132)	0.8 (178)	1.0 (224)	0.9 (127)	0.7 (124)
5 (most deprived)	1.1 (132)	1.1 (136)	1.1 (127)	1.3 (154)	1.2 (229)	1.1 (208)	1.0(142)	1.0 (162)
Missing IMD score	*0*	*3*	*5*	*2*	*11*	*7*	*7*	*4*

Proportion of people with undelivered mail and IMD quintile.

	**Trial 1 (N=163,525)**		**Trial 2 (N=150,417)**		**Trial 3 (N=265,434)**		**Trial 4 (N=168,480)**	
	Standard + Gist	Standard	Standard+ Narrative	Standard	Standard with GP endorsement	Standard	Standard with Enhanced Reminder	Standard
	% (n)	% (n)	% (n)	% (n)	% (n)	% (n)	% (n)	% (n)
**IMD quintiles**								
1 (least deprived)	0.4 (75)	0.4 (71)	0.4 (75)	0.4 (69)	0.3 (88)	0.3 (97)	0.3 (45)	0.4 (70)
2	0.5 (93)	0.5 (94)	0.5 (88)	0.4 (77)	0.3 (107)	0.4 (120)	0.4 (60)	0.3 (66)
3	0.4 (72)	0.6 (94)	0.5 (80)	0.5 (73)	0.5 (130)	0.4 (122)	0.5 (77)	0.4 (76)
4	0.6 (89)	0.5 (65)	0.7 (95)	0.6 (87)	0.5 (122)	0.6 (138)	0.7 (98)	0.4 (70)
5 (most deprived)	0.7 (88)	0.6 (78)	0.8 (90)	0.6 (68)	0.6 (115)	0.5 (102)	0.6 (88)	0.4 (71)
Missing IMD score	*1*	*2*	*0*	*3*	*4*	*2*	*1*	*5*

GP endorsement trial status cross-tabulated by enhanced reminder trial status

ER trial status	GP endorsement trial population			Total
	Usual letter	GP endorsement letter	Not in GP endorsement trial	
Not in ER trial	74200	66502	-----	140702
Control in ER trial	37880	30259	22274	90413
ER intervention in ER trial	21931	34662	21474	78067
Total	134011	131423	43748	309182
